# WikiBuild: A New Application to Support Patient and Health Care Professional Involvement in the Development of Patient Support Tools

**DOI:** 10.2196/jmir.1961

**Published:** 2011-12-08

**Authors:** Patrick Michel Archambault

**Affiliations:** ^1^Centre de santé et de services sociaux Alphonse-Desjardins (Centre hospitalier affilié universitaire de Lévis)Lévis, QCCanada; ^2^Département de médecine familiale et de médecine d’urgenceFaculté de médecineUniversité LavalQuébec, QCCanada; ^3^Traumatologie – urgence – soins intensifsCentre de recherche FRSQ du CHA universitaire de QuébecQuébec, QCCanada; ^4^Division de soins intensifsDépartement d'anesthésiologieUniversité LavalQuébec, QCCanada

**Keywords:** Medical informatics, patient-centered care, wikis, collaborative writing applications, knowledge translation, patient and public involvement

## Abstract

Active patient and public involvement as partners in their own health care and in the development of health services is key to achieving a health care system that is responsive to patients’ needs and values. It promotes better use of the health care system, and improves health outcomes, quality of life and patient satisfaction. By involving patients and health care professionals as partners in the creation and updating of patient health support tools, wikis—highly accessible, interactive vehicles of communication—have the potential to empower users to implement these support tools in daily life. Acknowledging the potential of wikis, and recognizing that they capitalize on the free and open access to information, scientists, opinion leaders and patient advocates have suggested that wikis could help decision-making constituencies improve the delivery of health care. They might also decrease its cost and improve access to knowledge within developing countries. However, little is known about the efficacy of wikis in helping to attain these goals. There is also a need to know more about the intention of patients and health care workers to use wikis, in what circumstances and what factors will influence their use of wikis. In this issue of the Journal of Medical Internet Research, Gupta et al describe how they developed and tested a new wiki-inspired application to improve asthma care. The researchers involved patients with asthma, primary care physicians, pulmonologists and certified asthma educators in the construction of an asthma action plan. Their paper—entitled “WikiBuild: a new online collaboration process for multistakeholder tool development and consensus building”—is the first description of a wiki-inspired technology built to involve patients and health care professionals in the development of a patient support tool. This innovative study has made important contributions toward how wikis could be generalized to involve multiple stakeholders in the development of other knowledge translation tools such as clinical practice guidelines or decision aids. More specifically, Gupta et al have uncovered potential action mechanisms toward increasing usage of these tools by patients and health care professionals. These are decreasing hierarchical influences, increasing usability and adapting a tool to local context. More research is now needed to determine if the use of the resulting wiki-developed plan will actually be higher than a plan developed using other methods. Furthermore, there is also a need to assess the intention of participants to continue using wiki-based processes on an ongoing basis. It is in this dynamic and continuous retroaction loop that the support tool users—both patients and health care professionals—can adapt and improve the product after its real-life shortcomings are revealed and as new evidence becomes available. As such, a wiki would be more than a simple patient support development tool, but could also become a dynamic and interactive repository and delivery tool that would facilitate ongoing and sustainable patient and professional engagement.

Active patient and public involvement as partners in their own health care and in the development of health services is key to achieving a health care system that is responsive to patients’ needs and values [1-3]. It promotes better use of the health care system and improves health outcomes, quality of life, and patient satisfaction [4]. By involving patients and health care professionals as partners in the creation and updating of patient health support tools, wikis—highly accessible, interactive vehicles of communication—have the potential to empower users to implement these support tools in daily life [5].

Acknowledging the potential of wikis and recognizing that they capitalize on the free and open access to information, scientists, opinion leaders, and patient advocates have suggested that wikis could help decision-making constituencies improve the delivery of health care [6,7]. Wikis might also decrease the cost of health care [8] and improve access to knowledge within developing countries [6,9,10]. However, little is known about the efficacy of wikis in helping to attain these goals. There is also a need to know more about the intention of patients and health care workers to use wikis and in what circumstances, and what factors will influence their use of wikis [11]. An ongoing scoping review on the use of wikis and collaborative writing applications in health care will better identify the areas where further knowledge synthesis is needed and the areas where more primary research remains to be done [12].

In this issue of the *Journal of Medical Internet Research*, Gupta et al [13] describe how they developed and tested a new wiki-inspired application to improve asthma care. The researchers involved patients with asthma, primary care physicians, pulmonologists, and certified asthma educators in the construction of an asthma action plan. Their paper—entitled “WikiBuild: a new online collaboration process for multistakeholder tool development and consensus building”—is the first description of a wiki-inspired technology built to involve patients and health care professionals in the development of a patient support tool. The findings of this study will thus be an important addition to the cumulative evidence being synthesized in the ongoing scoping review [12].

Given the drive for more patient and public involvement in health care, finding effective ways to engage patients in decision making has become paramount [14]. For asthma patients, the use of an action plan—a document written by health care professionals to guide patients’ individual self-management of worsening symptoms—has been shown to significantly reduce hospitalizations, emergency room visits, and missed work or school, and to significantly improve quality of life [15]. However, in practice, these asthma action plans are not used, and uptake has been low by clinicians and patients alike. Most existing action plans have been developed by teams consisting exclusively of medical experts who have focused on the content of action plans without addressing ease of use and visual design factors. By involving patients in the development of patient information materials, more relevant information can be included that is better adapted to the local context and that better meets the needs of end users [16].

In developing this custom-built application to enable peer-to-peer editing of the visual characteristics of an asthma action plan, the authors highlight the importance of the visual design of patient support tools. The way information is transmitted to patients greatly influences their decisions. Thomas Goetz brilliantly illustrates this fact in a popular TED Talk available on YouTube [17]. In this video, he points to seminal research showing that a drug facts box—a simple 1-page summary of relevant drug information—improves consumers’ knowledge of prescription drug benefits and side effects [18]. The WikiBuild process proposes a bold new way of incorporating patients’ and professionals’ action plan design preferences with the intention of increasing its uptake.

Overall, the WikiBuild application surpassed the authors’ expectations of usability in many aspects. Almost all the participants contributed to the development of the tool using the new wiki application. Even though participants had incentives to contribute, this very high contribution rate compares very well with editing rates within well-known wikis such as Wikipedia [19]. In the end, most participants were satisfied with the final action plan, and few participants perceived interstakeholder group hierarchies. One of the basic philosophies supporting the use of wikis for collaborative work is that authors are equal and authority is generally disregarded, since each contribution is judged by its merit and not by the degree or title of its author. Equality between individuals is one of the basic characteristics of collaboration, and research has shown that collaboration is hindered by power differences based on gender stereotypes and social status [20,21].

The main limitation acknowledged by the authors for their wiki-inspired application was that options in the wiki site were predetermined, possibly limiting user creativity. This limitation was intended to focus participants’ attention on adapting the visual aspects. However, this constraint possibly limited participants’ capacity to collaboratively write the action plan content, a process that could also increase its relevance and usability. Recognizing this vast potential, other scientists are exploring wikis to involve patients in collaborative content writing [22].

Notwithstanding this limitation, this innovative study has made important contributions toward how wikis could be generalized to involve multiple stakeholders in the development of other knowledge translation tools such as clinical practice guidelines or decision aids. More specifically, Gupta and colleagues have uncovered potential action mechanisms toward increasing usage of these tools by patients and health care professionals. These are decreasing hierarchical influences, increasing usability, and adapting a tool to the local context.

More research is now needed to determine whether the resulting wiki-developed plan will actually be used more than a plan developed by other methods. Furthermore, there is also a need to assess the intention of participants to continue using wiki-based processes on an ongoing basis. It is in this dynamic and continuous retroactive loop that the support tool users—both patients and health care professionals— can adapt and improve the product after its real-life shortcomings are revealed and as new evidence becomes available. As such, a wiki would be more than a simple patient support development tool; it could also become a dynamic and interactive repository and delivery tool that would facilitate ongoing and sustainable patient and professional engagement.

In conclusion, Gupta and colleagues have shed new light on how wikis could engage patients and health care professionals in the creation and use of an asthma action plan. By doing so, they have also paved the way to further exploration of wikis for patient and health care professional involvement in the development of many other knowledge translation tools such as decision aids and clinical practice guidelines.
